# GAMUT: GPU accelerated microRNA analysis to uncover target genes through CUDA-miRanda

**DOI:** 10.1186/1755-8794-7-S1-S9

**Published:** 2014-05-08

**Authors:** Shuang Wang, Jihoon Kim, Xiaoqian Jiang, Stefan F Brunner, Lucila Ohno-Machado

**Affiliations:** 1 Division of Biomedical Informatics, University of California, San Diego, CA, 92093, USA; 2 Programme in Biomedical Informatics, University of Applied Sciences Upper Austria, Hagenberg 4232, Austria

## Abstract

**Background:**

Non-coding sequences such as microRNAs have important roles in disease processes. Computational microRNA target identification (CMTI) is becoming increasingly important since traditional experimental methods for target identification pose many difficulties. These methods are time-consuming, costly, and often need guidance from computational methods to narrow down candidate genes anyway. However, most CMTI methods are computationally demanding, since they need to handle not only several million query microRNA and reference RNA pairs, but also several million nucleotide comparisons within each given pair. Thus, the need to perform microRNA identification at such large scale has increased the demand for parallel computing.

**Methods:**

Although most CMTI programs (e.g., the miRanda algorithm) are based on a modified Smith-Waterman (SW) algorithm, the existing parallel SW implementations (e.g., CUDASW++ 2.0/3.0, SWIPE) are unable to meet this demand in CMTI tasks. We present CUDA-miRanda, a fast microRNA target identification algorithm that takes advantage of massively parallel computing on Graphics Processing Units (GPU) using NVIDIA's Compute Unified Device Architecture (CUDA). CUDA-miRanda specifically focuses on the local alignment of short (i.e., ≤ 32 nucleotides) sequences against longer reference sequences (e.g., 20K nucleotides). Moreover, the proposed algorithm is able to report multiple alignments (up to 191 top scores) and the corresponding traceback sequences for any given (query sequence, reference sequence) pair.

**Results:**

Speeds over 5.36 Giga Cell Updates Per Second (GCUPs) are achieved on a server with 4 NVIDIA Tesla M2090 GPUs. Compared to the original miRanda algorithm, which is evaluated on an Intel Xeon E5620@2.4 GHz CPU, the experimental results show up to 166 times performance gains in terms of execution time. In addition, we have verified that the exact same targets were predicted in both CUDA-miRanda and the original miRanda implementations through multiple test datasets.

**Conclusions:**

We offer a GPU-based alternative to high performance compute (HPC) that can be developed locally at a relatively small cost. The community of GPU developers in the biomedical research community, particularly for genome analysis, is still growing. With increasing shared resources, this community will be able to advance CMTI in a very significant manner. Our source code is available at https://sourceforge.net/projects/cudamiranda/.

## Background

MicroRNAs (miRNAs) are single-stranded, small non-coding RNAs that control the expression of gene [1]. Target genes are either degraded at the mRNA level or inhibited at the protein level. With its ability to modulate target genes, miRNA has been shown to be associated with pathogenesis of several diseases such as cancer, metabolic and neurodegenerative diseases, and heart disease, to name just a few [2]. For this reason, miRNAs are biomarker candidates for diagnosis [3] and prognosis, including treatment response [4]. Many efforts have been made to develop prediction algorithms to identify miRNA-mRNA interactions. Widely used tools for miRNA target predictions are DNA-microT [5], miRanda [6], PicTar [7], PITA [8], RNA22 [9], and TargetScan [10]. Although existing microRNA target prediction algorithms such as TargetScan, PicTar and DIANS-microT show high accuracy for plant microRNAs, they suffer from low sensitivities and specificities [11]. In contrast, miRanda achieved the highest sensitivity [12] among them for experimentally validated mammalian targets [1].

The miRanda algorithm detects potential microRNA target sites from genomic sequences in two steps. Firstly, miRanda carries out sequence alignment of query (miRNA) and reference (3UTR) sequences through dynamic programming local- alignment based on sequence complementarity. Then the minimum free energy (MFE) score is calculated for each selected high alignment miRNA-mRNA pairs. Lastly, targets exceeding predetermined threshold MFE scores are selected as potential targets. Although miRanda is accurate, it is slow. For example, it takes more than three hours to run 2,000 queries against 30,000 references on an Intel Xeon 2.4 GHz CPU and 96 GB memory. Due to the quadratic run time complexity of the underlying sequence alignment algorithm used in miRanda, there is a long computation time when comparing query sequences against a large amount of reference sequences. This is challenging to the investigator who wants to analyze multiple samples of microRNA sequence data. The researcher typically has to run a sequence of pre-target-prediction steps, including short-read alignment to both genome and other small RNAs, novel miRNA prediction, and statistical test for differential expression [13]. Each of these individual steps could take several hours. In miRanda, the total execution time split of miRanda for alignment vs. MFE determination is 85% vs. 15%, on average, although the correct ratio will vary depending on the number of queries, the user's choices of threshold values, and hardware specification. In this study, we focus on boosting sequence alignment in miRNA target prediction, which is a critical step in microRNA analysis.

Sequence alignment requires scoring of the similarity between a short query sequence against a set of reference sequences. While the global alignment performs end-to-end matching of two sequences, the local alignment aims to find the highest scoring alignment of sub-sequences of the query and the reference sequences [14]. Thanks to natural selection, most microRNA target recognition sites are well preserved during evolution, and the task of prediction falls in the latter category. For high accuracy, Smith-Waterman (SW) [15] is the most widely used algorithm for local alignment. As SW has time complexity *O*(*n*^2^) due to the underlying dynamic programming, it has been modified through the addition of heuristic techniques to accelerate run time, as in BLAST [16] and FASTA [17] algorithms, at the cost of decreased sensitivity. Recently, an alternative solution for high-accuracy and high-speed SW algorithm was introduced by leveraging Field Programmable Gate Array (FPGA) [18], SIMD (Single Instruction Multiple Data) parallelisation on CPU [19,20] and Graphics Processing Unit (GPU) [21-28]. Since the cost of FPGA is high and the performance of CPUs is limited by the power constraints [29], GPUs became a very powerful and cost-effective platform for highly demanding scientific computing [30] that previously could only be performed using a supercomputer. GPU utilizes graphical pipelines to perform computation in applications traditionally handled by the CPU. Their highly parallel structure makes them more effective than CPUs for a range of complex algorithms. GPUs have been increasingly used in biomedical informatics applications [21-28,31] physics [32] and other research areas [33,34]. These existing SW implementations for GPU are designed for long sequences, usually for proteins and not for DNA or RNA. Also their CUDA implementation focused on calculating alignment scores only, as their main goal is optimizing database query, not the alignment itself [25]. This additional step is achieved through a backtracking routine in the alignment matrix, with has an extra quadratic time complexity. However, microRNAs are very short (around 22bp) and the problem requires backtracking between query and reference pair. We believe ours is the first CUDA-implementation of the SW algorithm for short sequence alignment with extended heuristics to identify microRNA targets.

## Methods

### The miRanda algorithm

The first version of miRanda was published in 2003, and the results were validated with microRNA targets in Drosophila melanogaster [6]. The most recently release is v3.3a from August 2010, which is available as open-source under the General Public License (GPL) [35]. The algorithm consists of two main parts. First, highly complementary regions are identified by using a dynamic programming algorithm that is based on the modified SW algorithm. Moreover, some heuristic rules are included to improve the prediction accuracy, where the most important one is the position- specific weighting schemes, which indicate the importance of the microRNA seed region. For the identified target sites, the minimum free energy of the microRNA-mRNA duplex is calculated using folding routines from the Vienna RNA secondary structure-programming library (RNAlib) [36-38]. Predictions that pass both thresholds of alignment score and minimum free energy are reported as possible target sites.

*Modified Smith-Waterman algorithm: *miRanda is based on a modified SW algorithm. We remind the readers of its main characteristics next so that they can follow the same notation in the CUDA section.

First, let us define the following notations.

• *q_k _*and *r_l _*are the *k*-th query and *l*-th reference sequences with length $L_{k}^{q}$ and $L_{l}^{r}$, respectively.

• *D_i,j _*is the alignment score of the base pair *q_k_*(*i*) and *r_l_*(*j*) at the position *i *of *q_k _*and the position *j*of *r_l_*, respectively, where $0\leq i<L_{k}^{q}$ and $0\leq j<L_{l}^{r}$.

• *A_i,j _*is a partial alignment score for diagonal continuation.

• *B_i,j _*is a partial alignment score for horizontal gap extension.

• *C_i,j _*is a partial alignment score for vertical gap extension.

• *d*(*q_k_*(*i*), *r_l_*(*j*), *i*) is the scoring function for match or mismatch, where *i *indicates whether the location is within the seed region or not.

• *O_i _*is the cost for opening a gap, where its value depends on whether *i *is within the seed region or not.

• *e_i _*is the cost for extending a gap, where its value depends on whether *i *is within the seed region or not.

where the seed region is defined as 2 <*i *< 8 by default.

Then, the boundary conditions for alignment scores are defined as *D*_0,*l *_= *D*_*k*,0 _*A*_0,*l *_= *A*_*k*,0 _= *B*_0,*l *_= *B*_*k*,0 _= *C*_0,*l *_= *C*_*k*,0 _= 0. Given the boundary conditions, the alignment scores can be updated as

(1) $$D_{i,j}=\text{max}\left\{\begin{array}{c} A_{i,j}\\ B_{i,j}\\ C_{i,j} \end{array}\right)$$

(2) $$A_{i,j}=\text{max}\left\{\begin{array}{c} 0\\ D_{i-1,j-1}+d\left(q_{k}\left(i\right),r_{l}\left(j\right),i\right) \end{array}\right)$$

(3) $$B_{i,j}=\text{max}\left\{\begin{array}{c} A_{i,j-1}+o_{i}\\ B_{i,j-1}+e_{i} \end{array}\right)$$

If *i *is in the seed region (e.g., 2 <*i *< 8 by default), *C*_*i,j *_= -1, otherwise

(4) $$C_{i,j}=\text{max}\left\{\begin{array}{c} A_{i-1,j}+o_{i}\\ C_{i-1,j}+e_{i} \end{array}\right)$$

*Specific scoring matrix: *The scoring function *d*(*q_k_*(*i*), *r_l_*(*j*),*i*) in miRanda algorithm is defined by two specific scoring matrices with following heuristic rules. Since target recognition between microRNA and mRNA is the end-goal, the complementary matching is imposed in miRanda, where the default value for A-U or G-C pairs is +5. Moreover, for G:U wobble pairs, a positive score is set to +1. All other nucleotide pairs have a default score of −3 (see Table 1). According to the observations of known microRNA targets, all scores and penalties have a higher weight in the microRNA seed region (default positions from 2 to 8), where a default scaling factor is set to 4. In addition, the positions 1, $L_{i}^{q}-1$ and $L_{i}^{q}$ are always scored 0, where $L_{i}^{q}$ is the *i*-th microRNA query length.

**Table 1:** Default miRanda scoring function.

	A	C	G	T/U
A	-3			
C	-3	-3		
G	-3	5	-3	
T/U	5	-3	1	-3

In the microRNA seed region, all vaules are multiplied by a scaling factor (the default is 4).

*Alignment score thresholding: *In miRanda, instead of just reporting the best match, all alignments between the microRNA query and the reference that exceed a given score threshold will be considered as candidate outputs, where the threshold is set to 140 by default. When two duplexes share matched region longer than six nucleotides, then miRanda will discard the one with smaller matching score. This criteria is achieved through sorting by the scores as primary key and the end positions as secondary keys of all alignments followed by the overlap comparisons among the sorted candidates. In the miRanda algorithm, the *n*-th valid alignment $K_{q_{k},r_{l}}^{n}$ between query *q_k _*and *r_l _*is represented by a tetrad $K_{q_{k},r_{l}}^{n}=\left\{D_{i*,j*},n,i*,j*\right\}$, where i*, j* are the end positions of query and reference, respectively, and *D*_*i,j *_are the corresponding alignment scores. *Backtracking: *miRanda provides both alignment scores and results obtained from the backtracking routine as output, together with the energy calculation. The idea of backtracking for an alignment $K_{q_{k},r_{l}}^{n}=\left\{D_{i*,j*},n,i*,j*\right\}$ is that the algorithm looks backward starting at the location (i*, j*) in the alignment score matrix *D *until reaching the termination criteria (i.e., $D_{i^{\prime },j^{\prime }}=0$), where the direction of a backward move follows the path in which the score matrices *B, C *and *D *are built up, as illustrated in equations 1 to 4. In the backtracking algorithm, an up move (i.e., (*i *- 1, *j*)) or a left move (i.e., (*i, j *- 1)) corresponds to a gap insertion in the query or reference, respectively. Besides, a diagonal move (i.e., (*i *- 1, *j *- 1)) indicates a match between reference and query at corresponding locations. The implementation of backtracking routine will be illustrated in details in the GPU implementation section.

*Free energy calculation: *For the identified target sites, the minimum free energy of the micro RNA-mRNA duplex is calculated using folding routines from the Vienna RNA secondary structure programming library (RNAlib) [36]. An energy threshold can be set for further filtering potential target sites, where only a prediction that passes both alignment score and energy thresholds is reported as an output. In the current version of CUDA-miRanda, the RNAlib is also included for completeness, but it has not been implemented on GPU. This is because the energy calculation function is not the bottleneck of the miRanda algorithm (e.g., it takes less than 15% of the total execution time in the worst cases). Moreover, the energy calculation function is implemented in a recursive way, which is not suitable for General-Purpose computing on Graphics Processing Units GPGPU on NVIDIA's Fermi GPUs. With the recently release of NVIDA's Kepler GPU, dynamic kernel execution and recursion are partially supported in the hardware. Although it would be interesting to accelerate the energy calculation on GPU, we will leave this for future work. In this article, we focus on the GPU implementation for the alignment step.

*Evolutionary conservation: *miRanda applies predefined thresholds to select miRNA-target pairs having high alignment score and low minimum free energy of the duplex structure. Among these, miRanda keeps the miRNA-target pairs that are also found in other species. More specifically, the hits found in *D. melanogaster *are considered conserved if they are also found in *D. pseudoobscura *and *A. gambiae*. However, it has been reported that about half of the predicted miRNA target sites in human are not conserved in other organisms based on the experimentally validated data [39]. Also, the original miRanda paper, which focused on the analysis of D. *melanogaster*, has reported that its conservation rate with A. *gambiae *is only 60%. This version of CUDA-miRanda did not include the scoring of conservation.

## GPU implementation of the miRanda algorithm

### GPGPU using CUDA

In GPU computing, CPU and GPU are used together in a heterogeneous co-processing computing model. Computationally intensive parts can often be accelerated by parallelization. This part of code is applied to the GPU while the sequential part of the application runs on the CPU. With the availability of the Compute Unified Device Architecture (CUDA) by NVIDIA, developers can now write code for both a CPU and a GPU in a similar way by using the instruction set of CUDA [40]. With CUDA, developer can bypass the need of direct working with low- level graphics API, so that GPU can be programmed using a high-level programming language for general purpose computing. In CUDA, the GPU is viewed as a computing device with massively parallel capability (e.g., supporting tens of thousands of concurrent threads). Moreover, the GPU (i.e., device) can be used as a coprocessor to the main CPU (i.e., host), where both host and device have their own dedicated memory space (i.e., host and device memory, respectively). The data transfer between host and device is achieved through the optimized API calls using the PCI-E bus.

**Figure 1:**
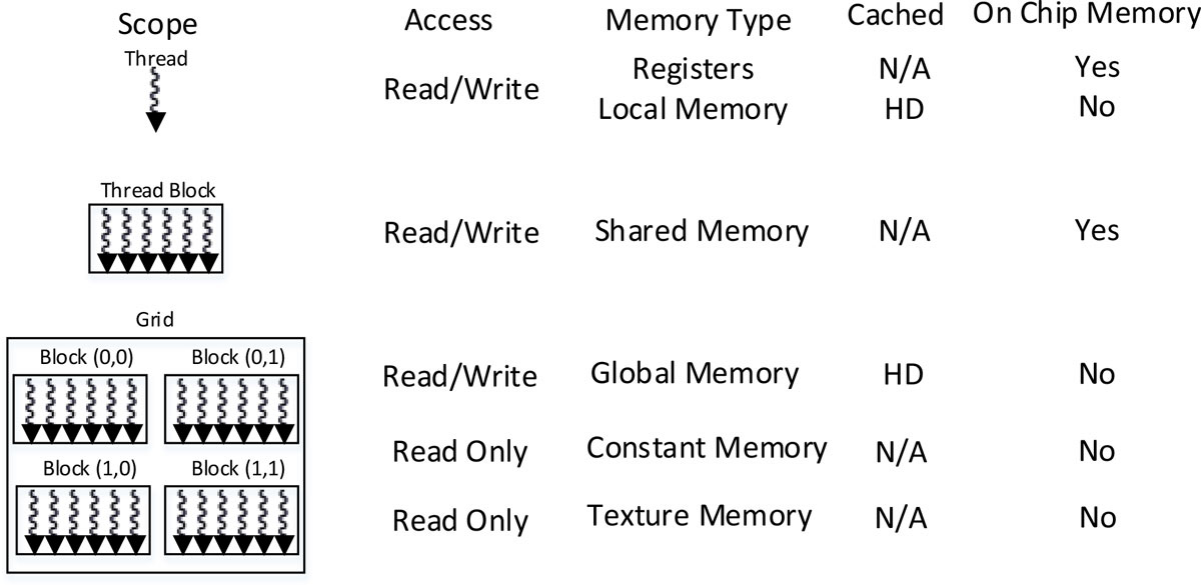
**CUDA thread and memory hierarchy**. Here, N/A and HD stand for not applicable and hardware dependent, respectively.

In CUDA, the thread hierarchy within a kernel is organized by the thread, the block of threads and the grid of blocks as shown in Figure 1, where different thread hierarchies have different memory scopes. An important best practice when programming CUDA is to make efficient use of various types of memory to maximize the memory throughputs. The device memory can be categorized as read/write per-thread registers and local memory, read/write per-block shared memory, read/write per-program global memory, read-only per-program constant memory and texture memory. In NVIDIA's GPU, registers and shared memory are embedded on the chip, which offers the fastest memory access. Moreover, threads within a block can communicate with each other by using the fast shared memory. In contrast, local and global memory are off-chip, where the accesses of such types of memory are very expensive (e.g., about 400 clock-cycle latency). However, such latency can be minimized by carefully scheduling the CUDA thread execution (e.g., assign sufficient arithmetic operations before/after each global memory transition) and following the most optimal access pattern [40]. Although the read-only constant and texture memories are also off-chip, the built-in caches can efficiently hide the latency, as long as the most memory accesses are within the cached memory locations. In addition, to achieve the best performance, branch in the program and bank conflicts in shared memory within a warp should be kept to a minimum, where a warp is a group of 32 parallel threads that execute the same instruction at a time.

#### CUDA Implementation of the modified SW algorithm

First, all the query and reference sequences are loaded from the input files in fasta format. In the preprocessing step, the queries and references are sorted by the length, so that tasks within a block have roughly equal complexity. Moreover, the original row-wise sequences are also stored into an optimized column-wise data structure in texture memory, by which sequence data can be cached and sequentially read by 32 threads in a warp within a single memory transaction. Moreover, instead of only reading a single nucleotide (i.e., 32 bytes per transaction), each thread reads a group of four nucleotides stored in a *cha *4 type, which results in a bandwidth of 128 bytes per transaction. Such procedure can significantly increase the memory throughput and reduce the number of accesses to the global memory.

**Figure 2:**
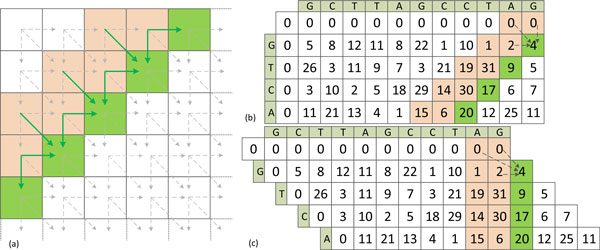
**Different representations of the alignment table**. (a) Original representation of the alignment table, where the update of each cell only depends on its upper, left and diagonal neighbors; (b) an example of the original representation of the alignment table; (c) the row shifting representation of the alignment table in the anti-diagonal direction to fit contiguous memory blocks and avoid bank conflicts.

Understanding the data dependencies in SW algorithm is another key factor for designing the parallelization. As shown in Figure 2(a), the update of each cell only depends on its upper, left and diagonal neighbors according to the update rules described in equations 1 to 4. Therefore, cells on the same anti-diagonal direction can be updated in parallel. For CUDA-miRanda, the maximum query length is set to 31, which is sufficient to fit the lengths of all microRNAs in our problem. Moreover, to improve the overall performance, memory accesses should be coalesced, which means a higher memory throughput can be achieved when memory access is consecutive and bank conflicts during shared memory access are avoided. To this end, a row shifting representation of the alignment table is used in our implementation as illustrated in Figures 2(b) and (c), where the *i*-th row is shifted to the left by *i *cells. Then, by observing the dependencies, for the calculation of alignment score *D *in the current column (as indicated in green in Figure 2(c)), at most two preceding columns (as indicated in orange in Figure 2(c)) are needed. Moreover, for the update of partial alignment scores *A, B *and *C*, only one preceding column is required. In our implementation, these intermediate scores are mapped to the fast shared memory instead of the global memory to reduce the memory access latency.

To enable traceback, it is necessary to store the whole path (i.e., backtracking matrix) from which the alignment scores are calculated. Thus, the backtracking matrix with size (*N_q _*× *N_r _*× *L*^q* ^× *L*^r* ^× s*izeo *(*track*)) is the most memory consuming part in CUDA-miRanda, where N_q_, N_r_, L^q* ^and *L*^r* ^are the number of queries and references, and the maximum length of queries and references, respectively. Moreover, s*izeo *(*track*) is the number of bits that are required to represent the backtracking path in each cell. It turns out that a large number of *N_q _*and *N *- *r *is desired for maximizing the parallelization, and *L*^q* ^and *L*^r* ^are data dependent. Therefore, to minimize the memory usage, we need to optimize the number of bits used for representing the *track *information. In our implementation, each *track *information is represented by 4 bits as follows:

• If the current cell is the starting point, the lowest 2 bits of *track *can represent the step of backtracking path as 0 for the end of backtracking, 1 for diagonal, 2 for up and 3 for left. And the same applies to the case when previous move is also from a diagonal direction.

• If the previous move is from a horizontal direction, the third bit of *track *can represent the step of backtracking path as 0 for diagonal and 1 for left.

• If the previous move is from a vertical direction, the fourth bit of *track *can represent the step of backtracking as 0 for diagonal and 1 for up.

Here, we introduce notations to be used in later formula. '*T*_1:2 _= *track *&*mask*_1_' extracts the lowest 2 bits from *track *variable, where *mask*_1 _= 3 and '&' is a bitwise *and *operation. '*T*_3 _= (*track *>> 2) &*mask*_2_' and '*T*_4 _= (*track *>> 3) &*mask*_2_' extract the third and forth bits from *track *variable, where *mask*_2 _= 1 and '>>' is a left bit-shift operation.

The rules for filling the backtracking array are as follows:

(5) $$T_{1:2}=\left\{\begin{array}{c} 0\;if\;D_{i,j}==0\\ 1\;if\;D_{i,j}==A_{i,j}\\ 2\;if\;D_{i,j}==B_{i,j}\\ 3\;if\;D_{i,j}==C_{i,j} \end{array}\right)$$

(6) $$T_{3}=\left\{\begin{array}{c} 0\;if\;C_{i,j}==C_{i-1,j}+e_{i}\\ 1\;else \end{array}\right)$$

(7) $$T_{4}=\left\{\begin{array}{c} 0\;if\;B_{i,j}==B_{i,j-1}+e_{i}\\ 1\;else \end{array}\right)$$

Therefore, in the proposed representation, the *size of *(*track*) is equal to 0.5 byte (i.e., 4 bits) and the backtracking matrix with 128 queries of length 32 and 100 references of length 30K only requires 128 × 100 × 32 × 30,000 × 0.5 bytes (i.e., 5860 MB), which can be easily fitted into a Tesla GPU with 6GB memory. For a dataset with a large number of queries and references, we can split the dataset into multiple batches, where each batch can be executed sequentially on GPU. We use an unsigned short type with 16 bits to store the track information of a group of four cells, so that we can improve the memory throughput when reading and writing the backtracking matrix. Moreover, as illustrated in the *Alignment Score Thresholding *section, there are multiple candidate alignments for each query and reference pair. The starting positions for both query and reference, the alignment score, and the index of each candidate are stored in an *ushort*4 type array in the global memory.

**Figure 3:**
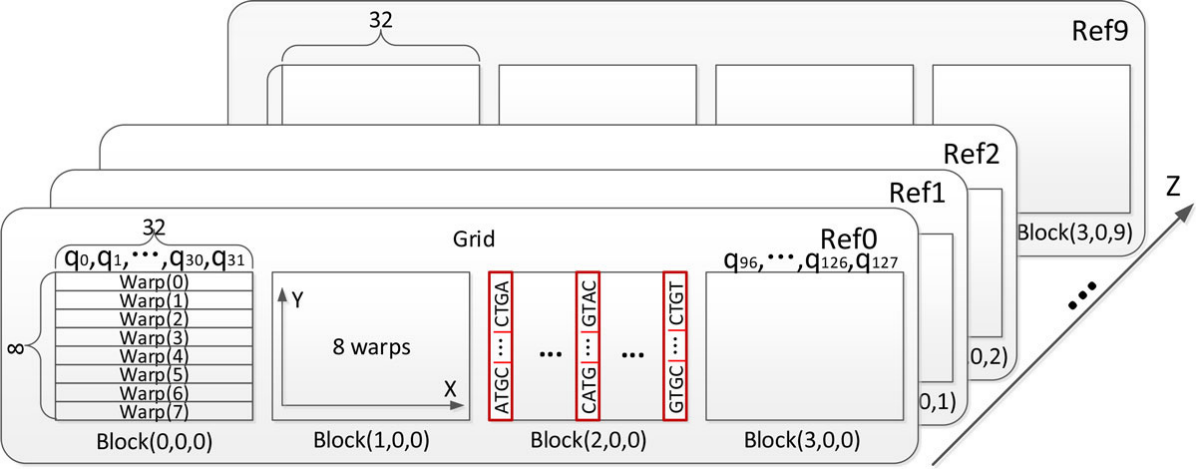
**The organization of blocks in the modified SW alignment function**. The dimensions of a block and a grid is 32 × 8 × 1 and $\left(\left\lfloor \frac{N_{q}-1}{32}\right\rfloor +1\right)\times 1\times N_{r}$, respectively. Here, the ⌊*⌋ function takes the largest integer that is less than *, and *N_q _*and *N_r _*are the number of queries and references that can be fitted in device memory, respectively. For example, *N_q _*= 128 and *N_r _*= 9 in this case.

**Figure 4:**
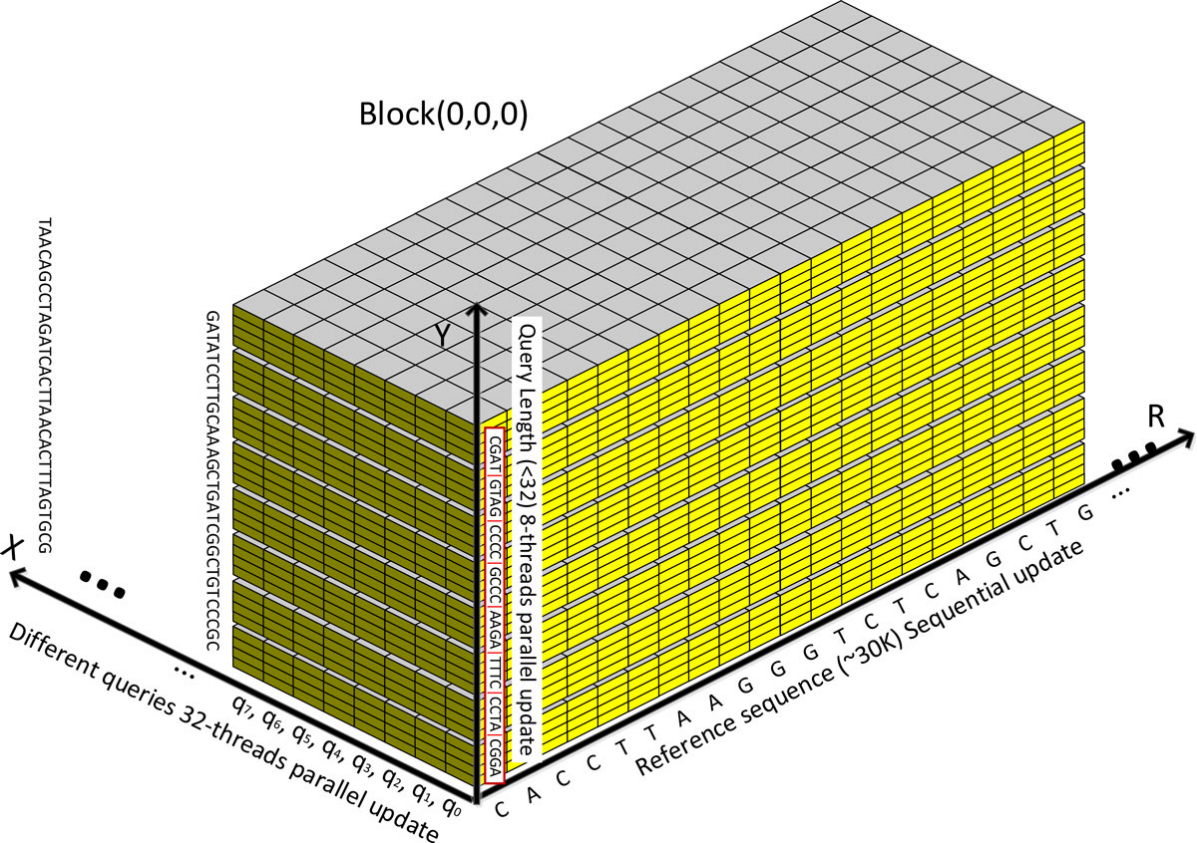
**Organization of threads in a block of the modified SW alignment function**. The modified SW algorithm is performed for a group of 32 query sequences in the *x *direction in parallel. As the query length is limited up to 32 nucleotides, 32 independent cells can be calculated at a time using 8-thread parallel update of groups of 4 nucleotides in the *y *direction. The above thread organization scheme makes sure that all 32 threads with in the same warp can operate on the same nucleotide location, which is able to minimize the intra-warp branch and bank conflict in shared memory.

**Figure 5:**
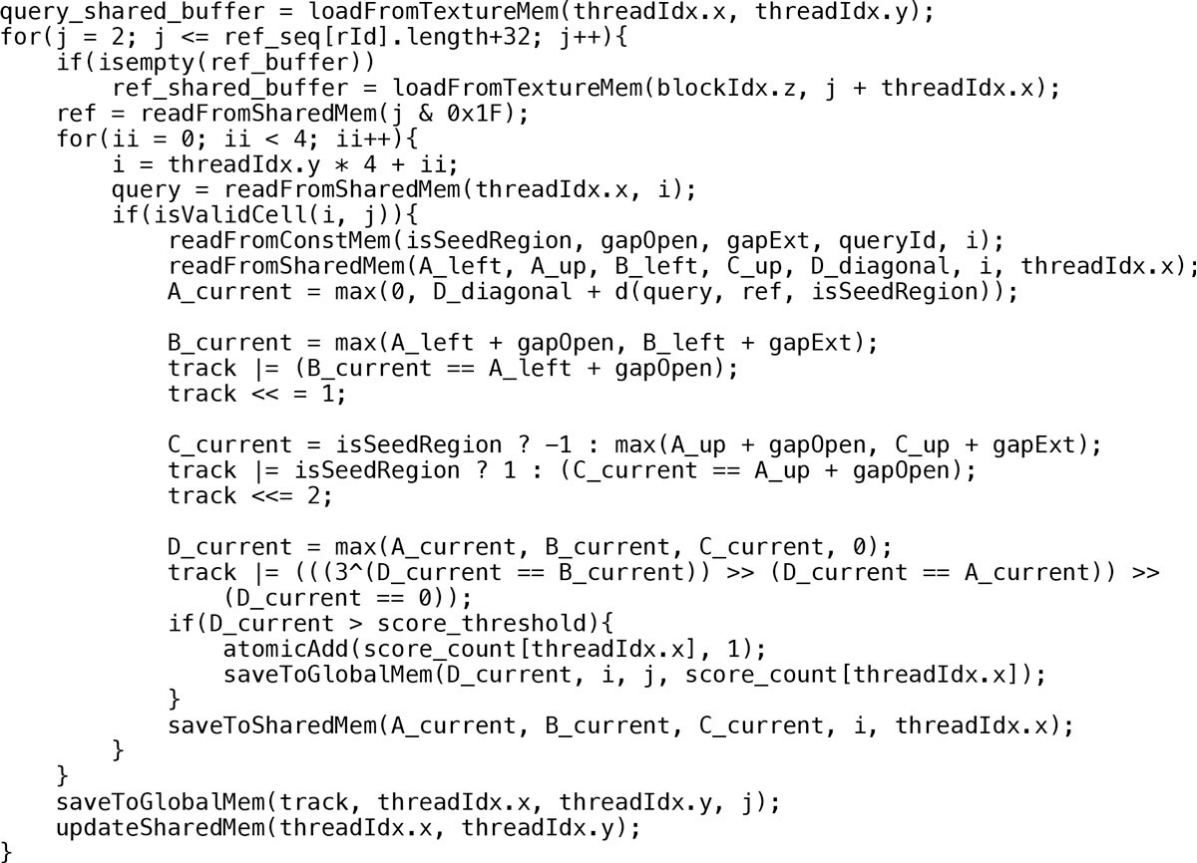
**Pseudocode for the modified SW alignment kernel**.

The organization of blocks in the modified SW alignment kernel is shown in Figure 3, where the dimensions of a block and a grid are 32 × 8 × 1 and $\left\lfloor \frac{N_{q}-1}{32}-1\right\rfloor \times 1\times N_{r}$, respectively. Here, ^* ^function takes the largest integer that is smaller than *, and *N_q _*and *N_r _*are the number of queries and references that can be fitted in the device memory, respectively. Moreover, the organization of threads in each block is shown in Figure 4. The modified SW algorithm is performed for a group of 32 query sequences in the *x *direction in parallel. As the query length is limited up to 32 nucleotides, 32 independent cells can be calculated at a time using 8-thread parallel update of groups of 4 nucleotides in the *y *direction. The above thread organization scheme makes sure that all 32 threads within the same warp can operate on the same nucleotide location, which enables minimization of the intra-warp branch and bank conflict in shared memory. The pseudocode of the modified SW alignment kernel is shown in Figure 5. As mentioned in *the miRanda Algorithm *section, the gap opening and extension costs, scoring matrices and alignment score update are different for the inside and outside seed regions. Moreover, the miRanda algorithm is based on the reversed order of each query. Therefore, the start and end positions for query sequences with different lengths are not the same. In our implementation, we pre- calculate the seed region for each query and store such information in a 32 bit *int *type array in the constant memory, as the length of the microRNA is bounded by 32 in our problem. Additionally, a value of 1 at a given bit indicates that the position is in the seed region. Moreover, the function *isValidCell*(*i, j*) in the kernel function is used to check the boundary condition in the row shifting representation. Finally, the *if *conditions in equation 5 are replaced through carefully designed bit and shift operations to avoid the branch within a warp as shown in Figure 5.

**Figure 6:**
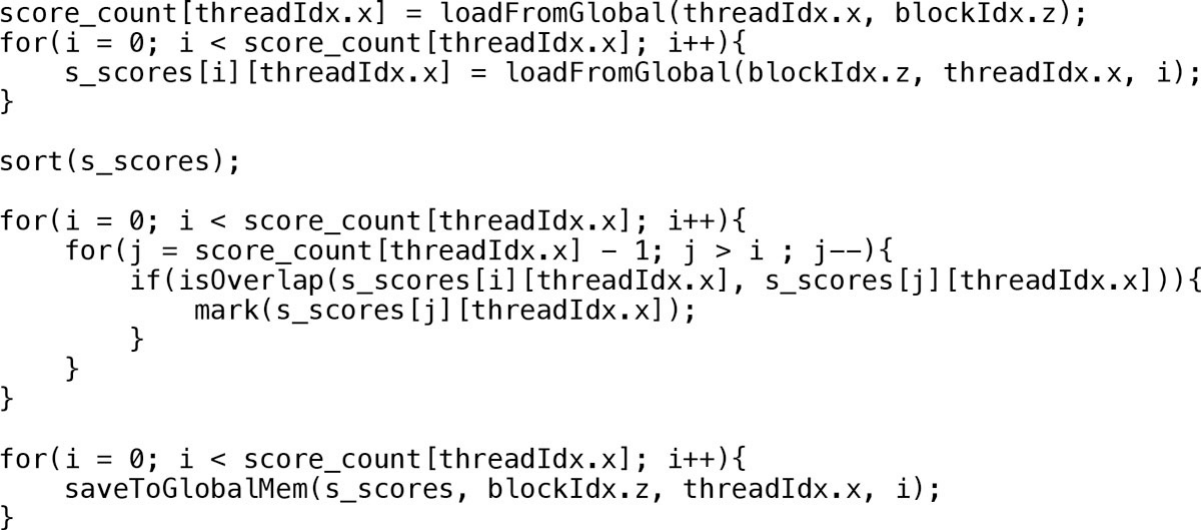
**Pseudocode for the score update kernel**.

The second stage of the CUDA-miRanda algorithm is to filter out the invalid alignment candidates as shown in Figure 6. The second kernel function uses almost 48 KB shared memory, which is used to allow the fast storing of candidates. For a single candidate, the data type *ushort*4 with 8 bytes is used to store score, index, and the alignment positions for query and reference sequences. As the shared memory size is 48 KB and we have reserved 128 bytes (i.e., 32 *queries *× 4 *bytes*) for the counts of a candidate, the maximum number of candidates per query-reference pair is bounded to 191 and can be calculated as follows:

(8) $$\left\lfloor \frac{48KB-128B}{sizeof\left(ushort4\right)\times 32\;queries}\right\rfloor =191$$

Subsequently, the parallelism is limited by the number of query and reference sequences, where the dimensions of a block and a grid are 32 × 1 × 1 and $\left(\left\lfloor \frac{N_{q}-1}{32}\right\rfloor +1\right)\times 1\times N_{r}$, respectively. The step to filter candidate alignments is straightforward, as it is inherited from miRanda and applied to our data structures.

**Figure 7:**
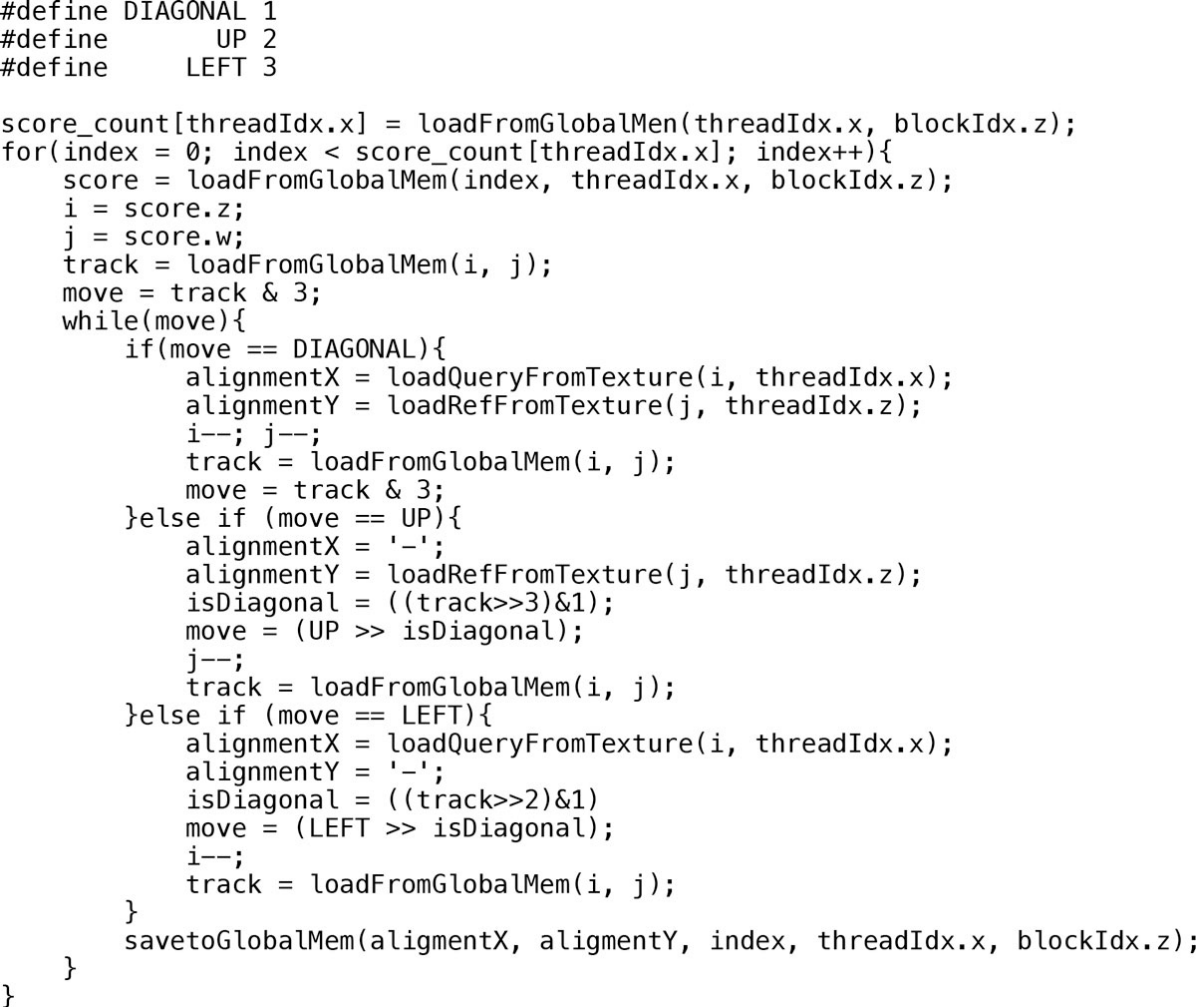
**Pseudocode for the backtracking kernel**.

Blazewicz *et. al*. [25] were the first to perform the backtracking procedure inside a kernel function. Unlike most other SW alignment algorithms, where only one alignment needs to be reported, in CUDA-miRanda, the backtracking of multiple alignments is desired for each query and reference pair. Moreover, in our implementation, we proposed a new backtracking strategy, which has taken the heuristic rules in the miRanda algorithm into account. The third stage of CUDA-miRanda algorithm for backtracking is illustrated in Figure 7. The logic and the workflow of backtracking were discussed in detail at the beginning of this subsection.

## Results

### Experimental setup

*Hardware: *All tests were performed on a 64-bit Linux workstation having two Intel Xeon E5620@2.4 GHz, 96GB RAM and four NVIDIA Tesla M2090 GPUs with 6 GB RAM each without ECC configuration.

*Test data: *To test the performance with as most application-related data as possible, real data sources for both queries (microRNA) and reference (3' UTRs) were used. MicroRNAs were downloaded from miRBase (databased release 18). FASTA files containing different numbers of known human microRNA sequences were created. These references comprise a set of 3' UTR sequence from http://www.targetscan.org (TargetScanHuman Release 6.1: March 2012). Only human transcripts were considered and brought into the FASTA file format so that they could be used as a reference input for miRanda. The raw reference dataset had 17,720 unique sequences and an average length of 1,167, ranging from 1 to 22,502. To test the performance in different conditions, we sampled multiple reference datasets with different average lengths from the Target Scan database.

*Parameter selections: *All test runs were performed using the default alignment parameters and the options *-quiet, -outfile, -keyval, -noenergy *unless specifically mentioned. This means that the output was configured to write only aligned query- reference pairs into a file in key-value format, where the I/O cost for outputting the results was not considered. All the results were obtained over an average of ten trials, and we have verified that the exact same targets were predicted in both CUDA- miRanda and the original miRanda implementations through multiple testing datasets. Finally, the energy calculation was deactivated for these tests, as the focus was on the most time consuming part, the alignment.

### Performance evaluation

In order to show the performance of CUDA-miRanda, the total execution time of the single and multi-GPU setups were compared to the original CPU-only version miRanda 3.3a on different datasets. CUDA-miRanda emerged from a specific use case where targets with hundred putative novel microRNA sequences should be predicted. Due to the parallel grouping of microRNA sequences in warps, the best alignment performance can be achieved for multiples of 32 queries. Table 2 presents the total execution times for various query sizes (i.e., 1, 32, 64, 128, and 256) against a reference dataset containing 256 references with the average length of 20K. References in the dataset are grouped by the reference batch size (RBS) of 32 in this test. For example, a dataset with 256 references and RBS = 32 will be split into 8 groups. The rightmost columns show the gained speed of CUDA-miRanda on 4 GPUs over the original miRanda on CPU. The single-GPU implementation can achieve a speed-up of more than 55x, and 2 and 4 GPUs setup can achieve a speed-up of 102x to 166x in the best cases. Moreover, we can see that the performance gain of CUDA-miRanda increases as the number of queries increases. This is because that the full computational power of GPU can be exploited only if there exists sufficient data that need to be executed. Finally, the scalability of the multi-GPU execution is also shown in Table 2, where the performance ratios of 4 vs. 1 and 2 vs. 1 GPUs are 2.98 and 1.83, respectively. The performance loss in scalability is due to the communication and scheduling overhead between host and devices.

**Table 2:** Execution time in seconds for various numbers of microRNA sequences against a reference dataset.

# of queries	1 GPU		2 GPUs		4 GPUs		miRanda on CPU	4GPUs vs. CPU
	
	Time	GCUPs	Time	GCUPs	Time	GCUPs	Time	Speedup
1	2.48	0.06	1.44	0.1	1.25	0.12	7	5x
32	2.59	1.8	1.92	2.44	1.39	3.38	177	127x
64	5.08	1.79	2.86	3.17	1.97	4.62	288	146x
128	9.89	1.79	5.58	3.17	3.63	4.89	580	159x
256	19.25	1.8	10.49	3.3	6.47	5.36	1076	166x

The reference dataset contained 256 sequences with average length of 20K. The orignial miRanda 3.3 on CPU and our proposed CUDA-miRanda on GPUs were compared.

**Figure 8:**
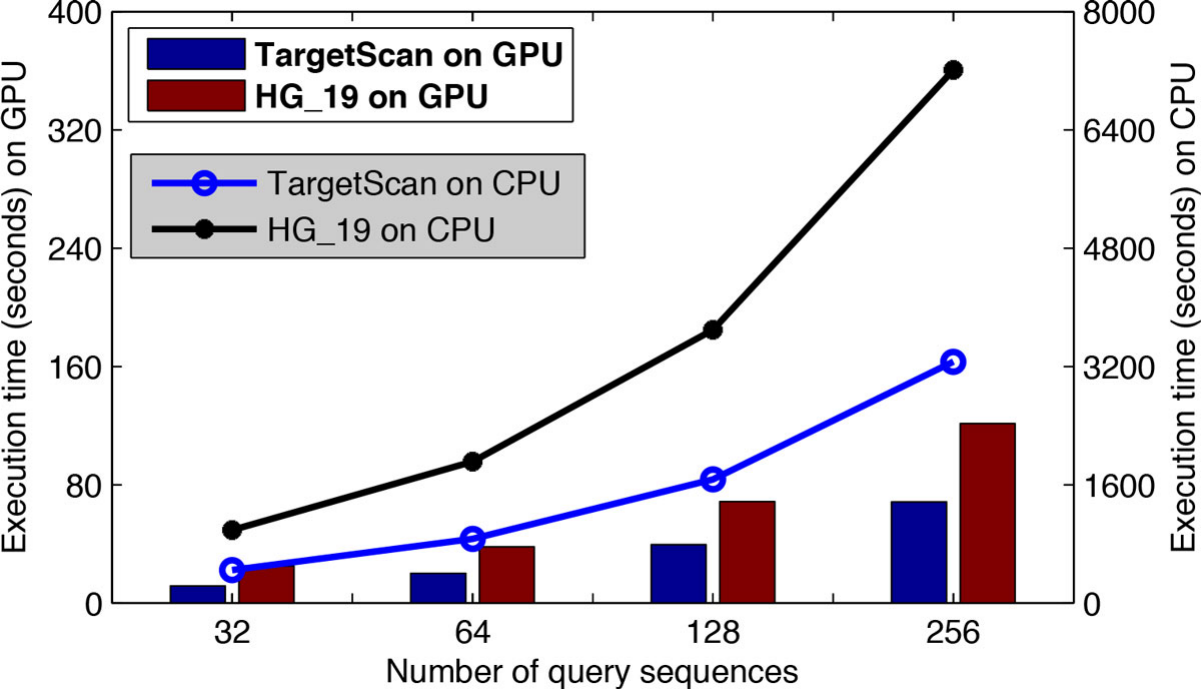
**Performance comparisons in terms of execution time (seconds) between CUDA-miRanda and miRanda**. We use Target Scan and HG_19 datasets with different number of queries.

The impact of different average reference lengths on GPU performance was also investigated and the results are shown in Table 3, where both the reference and query dataset contained 256 sequences. In Table 3, we can see that a longer average reference length in the dataset always showed a better performance of CUDA- miRanda in terms of both execution times and GCUPs. The speed-up of 4 GPUs vs. 1 CPU is also shown in Table 3 for reference. Moreover, we can see that the 4 GPUs performance is worse than that of 1 and 2 GPUs in the case of an average reference length of 1000. This is because the additional communication and scheduling overhead between host and devices overtake the performance gain by adding more computational resources for the limited computational tasks. This also demonstrated that the full computational power and the concealment of scheduling and communication overhead are only achieved if sufficient tasks are available on GPUs. Moreover, other two tests were performed to investigate the impact of different reference batch sizes (RBSs) on GPU performance in Tables 4 and 5, where 256 and 32 queries were used, respectively. For both tests, the size of the reference dataset was fixed at 256. Various settings for the reference batch size (i.e., 8, 16, 32 and 64) were used to analyze the impact on the algorithm performance. The resulting execution times and GCUPs are depicted in Tables 4 and 5. We can see that the GPU performance was stable for different RBS settings when sufficient number of queries (e.g., 256) was available in the test as shown in Table 4. In contrast, when only a limited number of queries (e.g., only 32) were available on the GPU, the GPU performance was sensitive to the selection of RBS. Table 5 showed that better performance could be achieved when a larger RBS was used. This also demonstrated that GPU is designed for computational intensive tasks, where full advantage in terms of speed is only achieved if sufficient data and operations can be fed into the GPU. Finally, we tested the proposed tools with an additional utr3prime_NM_hg19 data, which has 33,727 reference sequences with average length of 1361, ranging from 1 to 22,561. By using 256 test queries, CUDA-miRanda reduced the execution time from +120 minutes on a CPU to less than 2 minutes on GPUs as shown in Figure 8. Similarly, for the targetScan data with 256 input queries, the proposed CUDA- miRanda completes in about 1 minute on the GPU versus 1 hour on the CPU.

**Table 3:** Impact of different average reference lengths on performance.

Average Reference Length	1 GPU		2 GPUs		4 GPUs		miRanda on CPU	4GPUs vs. CPU
	
	Time	GCUPs	Time	GCUPs	Time	GCUPs	Time	Speedup
1000	1.55	0.99	1.42	1.08	1.6	0.96	54	33x
5000	4.83	1.59	3.17	2.43	2.57	3.01	240	93x
10000	8.8	1.72	5.19	2.92	3.66	4.14	556	151x
22000	19.25	1.8	10.49	3.3	6.47	5.36	1076	166x

Both reference and query datasets have 256 sequences, and the execution time was measured in seconds.

**Table 4:** The impact of different RBSs on the GPU performance.

RBS	1 GPU		2 GPUs		4 GPUs		miRanda on CPU	4GPUs vs. CPU
	
	Time	GCUPs	Time	GCUPs	Time	GCUPs	Time	Speedup
8	19.56	1.77	10.74	3.23	6.56	5.28	1076	164x
16	19.25	1.8	10.57	3.28	6.48	5.35		166x
32	19.25	1.8	10.49	3.3	6.47	5.36		166x

Both reference and query datasets have 256 sequences and the execution time was measured in seconds.

**Table 5:** Another sample table title.

RBS	1 GPU		2 GPUs		4 GPUs		miRanda on CPU	4GPUs vs. CPU
	
	Time	GCUPs	Time	GCUPs	Time	GCUPs	Time	Speedup
8	8.87	0.52	4.7	0.99	2.94	1.59	177	60x
16	4.49	1.04	2.48	1.88	1.62	2.88		109x
32	2.59	1.8	1.92	2.44	1.39	3.38		127x
64	2.57	1.82	1.63	2.87	1.27	3.68		139x

The impact of different RBSs on the GPU performance, where reference and query datasets have 256 and 32 sequences, respectively. Time was measured in seconds.

## Conclusion

Although there are many existing GPU-based implementations of the SW algorithm [21-28], most of them are focusing on the problem of database search with the report of the best matching score. The work reported in [25] is most relevant to our own, which takes the backtracking of pairwise alignments into account. However, all aforementioned implementations were unable to meet the high demands of the microRNA target identification tasks, which requires specific scoring schemes with heuristic rules, multiple alignment outputs within a single query and a reference sequence pair, alignment traceback, and a short query sequence against a long reference sequence alignment. Therefore, in this work, we have presented CUDA-miRanda, a fast microRNA target identification algorithm that takes advantage of the massively parallel computing on a GPU using CUDA. Speeds of more than 5.36 GCUPs were achieved on a server with 4 NVIDIA Tesla M2090 GPUs. In comparison with the original miRanda algorithm, evaluated on an Intel Xeon E5620@2.4 GHz CPU, the experimental results show up to 166 times performance gain in terms of execution times. The results demonstrate that massively parallel computing on GPU can significantly expedite studies of computational microRNA target identification. The gains in speed should translate into the completion of more experiments and potentially the acceleration of new discoveries. We are currently planning to use this implementation for the prediction of targets for differentially expressed microRNAs in children affected with Kawasaki disease and controls and have made our code and examples available so that others could use on their data as well. The cost of an entry-level GPU-enabled hardware to run these tests is around $1,000 today, a fraction of the cost that would take us to buy time on an HPC facility. For example, the current rental price of Cluster GPU (NVIDIA Tesla) is about $2.1 per hour. By including the provisioning and data transfer costs, one could conduct the same analysis with less than $20. Additionally, we have the advantage of keeping these human sequences private within our HIPAA-compliant environment.

## Abbreviations

CMTI, Computational microRNA target identification; SW, Smith-Waterman; GPU, Graphics Processing Units; CUDA, Compute Unified Device Architecture; GCUPs, Giga Cell Updates Per Second; HPC, high performance compute; MFE, minimum free energy; FPGA, Field Programmable Gate Array; SIMD, Single Instruction Multiple Data; GPL, General Public License; GPGPU, General-Purpose computing on Graphics Processing Units; RBS, reference batch size.

## Competing interests

The authors have received a gift from NVIDIA in the form of hardware: two NVIDIA Tesla C2070 GPUs and one Tesla K20c GPU.

## Authors' contributions

SW carried out the design and implementation of GPU-accelerated algorithm, conducted most of the analysis and drafted the manuscript. JK participated in the design and coordination, as well as contributed to draft the manuscript. XJ participated in the design of the study and helped to draft the manuscript. SFB prepared the data, performed preliminary studies and wrote portions of this manuscript. LOM provided oversight for this study and helped to edit the manuscript. All authors read and approved the final manuscript.

## Declarations

Publication for this article has been funded by the National Library of Medicine (R00LM011392) and the National Institutes of Health (U54HL108460).

This article has been published as part of *BMC Medical Genomics *Volume 7 Supplement 1, 2014: Selected articles from the 3rd Translational Bioinformatics Conference (TBC/ISCB-Asia 2013). The full contents of the supplement are available online at http://www.biomedcentral.com/bmcmedgenomics/supplements/7/S1.
